# SARS-CoV-2 infection: diagnostic testing results occasionally require special attention

**DOI:** 10.1080/22221751.2020.1814165

**Published:** 2020-09-06

**Authors:** Elisabetta Riva, Pier Paolo Sainaghi, Ombretta Turriziani, Guido Antonelli, Giuseppe Patti

**Affiliations:** a Virology Unit, Campus Bio-Medico University of Rome, Rome, Italy; b Department of Translational Medicine, University of Eastern Piedmont, Novara, Italy; c Microbiology and Virology Section, Department of Molecular Medicine, Sapienza University of Rome, Rome, Italy

**Keywords:** SARS-CoV-2 infection, diagnostic tests, SARS-CoV-2 serological tests, SARS-CoV-2 molecular tests, lack of antibodies

## Abstract

The case refers to a 51-year-old symptomatic man with a new SARS-CoV-2 RNA positive nasopharyngeal swab after two negative ones and the lack of significant development of antibody response measured by different diagnostic serological test. Our case underlines that a discrepancy between clinical course of SARS-CoV-2 infection and results from diagnostic tests may exist. This concept is rapidly emerging and supports the need for a deep knowledge of available and “in development” tests for a correct interpretation of their findings.

An immunocompetent 51-year-old healthcare worker developed high fever, cough and fatigue on March 17 (Time 0). A nasopharyngeal swab tested positive for Severe Acute Respiratory Syndrome-coronavirus-2 (SARS-CoV-2) RNA (Figure 1), after which the man was quarantined and treated at home with hydroxychloroquine. He had a rapid and full recovery. On days 29 and 31, two subsequent nasopharyngeal swabs tested negative for SARS-CoV-2 detection and he was readmitted to work. Two weeks later a serum sample was collected to detect antibodies against SARS-CoV-2, but no serum specific IgG antibodies were detected by chemiluminescence immunoassay (CLIA, LIAISON^TM^ SARS-CoV-2 S1/S2 IgG, DiaSorin). A repeated sample showed a very low IgG antibody positivity by enzyme-linked immuno-sorbent assay (ELISA, EDI™ Novel Coronavirus COVID-19 IgG, EDI-INC, Alifax, Index 1.4, cut-off 1.1, Target Nucleocapsid Protein), in the absence of IgM antibodies (ELISA, EDI™ Novel Coronavirus COVID-19 IgM, EDI-INC, Alifax, cut-off 1.1, Target Matrix Protein) . Thereafter, while asymptomatic, he was subjected to a new nasopharyngeal swab because of close contact with a positive healthcare worker. At this time, the swab was again positive with a low virus load, as suggested by the amplification of only one specific gene for SARS-CoV-2 (E gene – Ct 34.6, Gene S – Not Detected, RealStar^TM^ SARS-CoV-2 RT-PCR Kit 1.0, Altona). After one more week, the swab became negative without any change in antibody response pattern (by both CLIA and ELISA).

**Figure 1.**
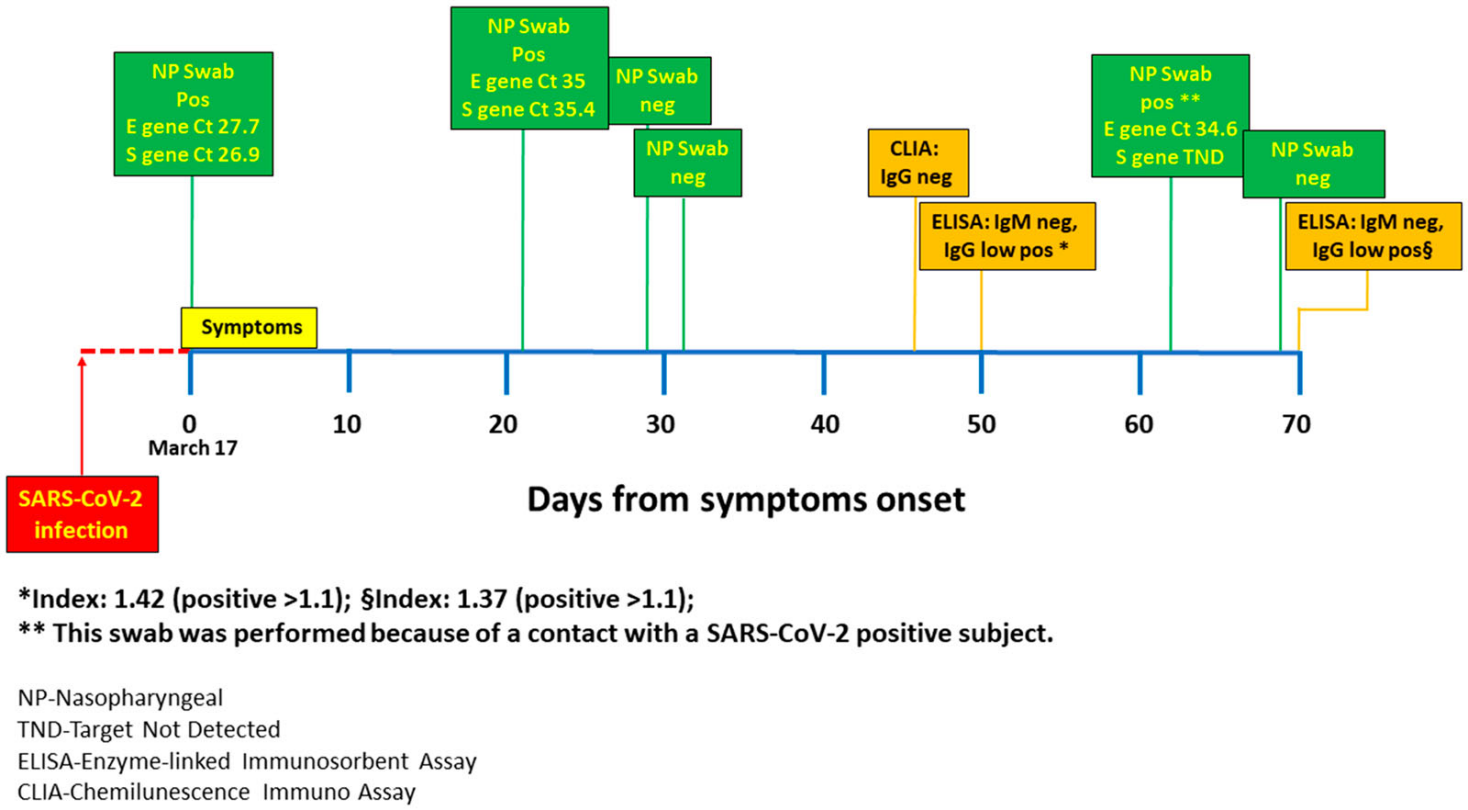
The image depicts the clinical course and results of laboratory tests over time.

The aim of this case report is to add a further contribution to the current discussion regarding the interpretation and clinical significance of results of currently available tests in diagnosing and monitoring SARS-CoV-2 infection. Our paper renews the need for careful interpretation of currently available tests.

Indeed: The use of serological tests may occasionally fail to detect subjects with a previous symptomatic infection. This may be due to different/low sensitivity of available tests in relation to: antigen used (for instance nucleocapsid vs spike); type of antibodies detected (IgG, IgM, IgA or total); concomitant therapies affecting the development of humoral response; individual immune response and different antibodies profiles in relation to the severity of the disease [1–4]. In our case, all these hypotheses are conceivable, because IgG antibodies were detected by ELISA (though with a very low positivity) but not by CLIA (i.e. the two tests target different antigens). Moreover, IgA antibodies were not tested and the patient was given hydroxychloroquine.

In addition, the detection of SARS-CoV-2 antibodies may not assure significant protection against either reactivation or re-infection, because it does not necessarily indicate the presence of protective antibodies. Therefore, the reason the swab retested positive after the resolution of the symptomatic infection and the consequent two negative swabs cannot be determined by performing only serological tests. Indeed, the patient did not develop a significant antibody response to SARS-CoV-2 infection despite it being clinically overt. It is worth nothing that the lack of significant antibodies response does not necessarily equal a lack of T-cell response, which might have been responsible for a prompt control of the second episode of infection/reactivation.

Molecular tests for SARS-CoV-2, even if in general sufficiently sensitive and specific, occasionally fail to properly screen infected patients because of false-negative or false-positive results.

False-negative results may be due to the extraction/Real Time-PCR workflow, inappropriate sample collection and/or low viral load in the sample. Sensitivity is a complex issue, as detection in the upper airways (nasopharynx and oropharynx) is affected by multiple factors, including duration of illness prior to testing and the limit of detection of the RT-PCR assay used. However, this was probably not the situation in our case, as two consecutive swabs were negative after the complete clinical resolution. False-positive tests can also occur, caused by technical errors or sample contamination. Notably, a positive molecular test indicates only the detection of viral RNA and may be unrelated to the presence of infectious virus. A few cases were previously reported positive after two consecutive negative swabs [5], but here attempts to isolate the virus in culture were unsuccessful [5–7]. This might reflect a lack of infectivity in the swab, but also the difficulty of achieving virus isolation in presence of a low viral load. This is a well-known and clear-cut concept in diagnostic virology. Thus, it is difficult to establish whether in our patient the swab was retested positive due to testing error, detection of viral RNA free-fragments persistent from the first infection, mild re-infection or an actual reactivation [7–10]. However, the presence of a positive swab (especially at low virus load) following two negative ones, in the absence of antibody detection or increase, does not necessarily indicate virus transmissibility; it requires confirmation and monitoring before drawing definite conclusions.

Given the dynamics of the infection and the host response, direct detection of the virus in the respiratory tract by PCR has been suggested as the optimal strategy for the initial diagnosis. Conversely, combining PCR and serological tests is deemed an appropriate approach for monitoring patients during the infection and checking their recovery [11]. We absolutely agree, but repeat that, as in our case, the latter approach might fail to correctly monitor the course of the infection or the patient’s recovery due to differences in sensitivity of the currently available tests and individual immuno-response.

Our case corroborates and strengthens other recent data [12], underlining and confirming that a discrepancy may exist between the clinical course of SARS-CoV-2 infection and results from laboratory tests. Thus, given the possible limitations of these tests, we cannot conclude that a sole “test-driven strategy” is always applicable; indeed, at least in some cases, a “test-driven” plus a “symptom-based” approach, along with a careful cases’ epidemiological history, should be used in managing exposed or infected people. Studies on the clinical performance of current and new SARS-CoV-2 tests are needed.

## References

[1] Liu Z-L, Liu Y, Wan L-G, et al. Antibody profiles in mild and severe cases of COVID-19. Clin Chem. 2020;66:1102–1104. doi: 10.1093/clinchem/hvaa137 32521002 PMC7314168

[2] Okba NMA, Müller MA, Li W, et al. Severe acute respiratory syndrome coronavirus 2-specific antibody responses in coronavirus disease patients. Emerg Infect Dis. 2020;6:1478–1488. doi: 10.3201/eid2607.200841 PMC7323511 32267220

[3] GeurtsvanKessel CH, Okba NMA, Igloi Z, et al. An evaluation of COVID-19 serological assays informs future diagnostics and exposure assessment. Nat Commun. 2020;11:3436. doi: 10.1038/s41467-020-17317-y 32632160 PMC7338506

[4] Rikhtegaran Tehrani Z, Saadat S, Saleh E, et al. Specificity and performance of nucleocapsid and spike-based SARS-CoV-2 serologic assays. medRxiv. 2020. doi: 10.1101/2020.08.05.20168476 PMC7605638 33137138

[5] Arnaout R, Lee RA, Lee GR, et al. SARS-CoV2 testing: the limit of detection matters. bioRxiv. 2020. doi: 10.1101/2020.06.02.131144

[6] Callahan C, Lee R, Lee G, et al. Nasal-swab testing misses patients with low SARS-CoV-2 viral loads. medRxiv. 2020. doi: 10.1101/2020.06.12.20128736

[7] Wölfel R, Corman VM, Guggemos W, et al. Virological assessment of hospitalized patients with COVID-2019. Nature. 2020;581:465–469. doi: 10.1038/s41586-020-2196-x 32235945

[8] Zhang B, Liu S, Dong Y, et al. Positive rectal swabs in young patients recovered from coronavirus disease 2019 (COVID-19). J Infect. 2020. DOI:10.1016/j.jinf.2020.04.023. Online ahead of print PMC7177113 32335176

[9] Xing Y, Mo P, Xiao Y, et al. Post-discharge surveillance and positive virus detection in two medical staff recovered from coronavirus disease 2019 (COVID-19), China, January to February 2020. Euro Surveill. 2020;25:2000191. doi: 10.2807/1560-7917.ES.2020.25.10.2000191 32183934 PMC7078824

[10] Chandrashekar A, Liu J, Martinot AJ, et al. SARS-CoV-2 infection protects against rechallenge in rhesus macaques. Science. 2020. DOI:10.1126/science.abc4776. Online ahead of print PMC7243369 32434946

[11] Sethuraman N, Jeremiah SS, Ryo A. Interpreting diagnostic tests for SARS-CoV-2. JAMA. 2020. DOI:10.1001/jama.2020.8259. Online ahead of print 32374370

[12] Green DA, Zucker J, Westblade LF, et al. Clinical performance of SARS-CoV-2 molecular testing. J Clin Microbiol. 2020. doi: 10.1128/JCM.00995-20. Online ahead of print PMC7383556 32513858

